# Comparison of Lumped Oscillator Model and Energy Participation Ratio Methods in Designing Two-Dimensional Superconducting Quantum Chips

**DOI:** 10.3390/e24060792

**Published:** 2022-06-07

**Authors:** Benzheng Yuan, Weilong Wang, Fudong Liu, Haoran He, Zheng Shan

**Affiliations:** State Key Laboratory of Mathematical Engineering and Advanced Computing, Zhengzhou 450001, China; benzhengyuan@outlook.com (B.Y.); wlwang19888@163.com (W.W.); lwfydy@126.com (F.L.); gary_hehaoran@163.com (H.H.)

**Keywords:** circuit quantization methods, superconducting quantum chips, circuit quantum electrodynamics, quantum information

## Abstract

Over the past two decades, superconducting quantum circuits have become one of the essential platforms for realizing quantum computers. The Hamiltonian of a superconducting quantum circuit system is the key to describing the dynamic evolution of the system. For this reason, various methods for analyzing the Hamiltonian of a superconducting quantum circuit system have been proposed, among which the LOM (Lumped Oscillator Model) and the EPR (Energy Participation Ratio) methods are the most popular ones. To analyze and improve the design methods of superconducting quantum chips, this paper compares the similarities and differences of the LOM and the EPR quantification methods. We verify the applicability of these two theoretical approaches to the design of 2D transmon quantum chips. By comparing the theoretically simulated results and the experimentally measured data at extremely low temperature, the errors between the theoretical calculation and observed measurement values of the two methods were summarized. Results show that the LOM method has more parameter outputs in data diversity and the qubit frequency calculation in LOM is more accurate. The reason is that in LOM more coupling between different systems are taken into consideration. These analyses would have reference significance for the design of superconducting quantum chips.

## 1. Introduction

Since quantum computing was proposed, many physical solutions have emerged to realize quantum computing, such as superconducting quantum circuits [1], photons [2], ion traps [3], semiconductor quantum dots [4], etc. Superconducting qubits stand out among many schemes due to their strong designability, ample controllable space, high scalability, and high compatibility with existing micro-nano processing technologies [5,6,7]. The study of superconductivity itself is of fundamental importance [8,9]. Moreover, it is also important in quantum computing, like realizing quantum gates [10,11], and quantum mechanical sensing [12]. For superconducting quantum circuit, the cavity quantum electrodynamics theory provides a theoretical basis [13,14], and circuit quantum electrodynamics systems have become one of the most promising platforms for realizing robust and scalable general-purpose quantum computers [15,16,17,18].

Circuit quantum electrodynamics system consists of two crucial parts: a high-quality superconducting microwave cavity and superconducting artificial atoms [19,20]. Artificial atoms are equivalent to non-harmonic circuits composed of Josephson junctions and capacitors in parallel [21,22]. So far, according to the ratio of Josephson junction energy $E_{j}$ to capacitance energy $E_{c}$, superconducting qubits can be divided into three categories: charge qubit (EjEjEcEc≪1), magnetic flux qubit (EjEjEcEc≈100), and phase qubit (EjEjEcEc≈104) [16,17,18]. Transmon qubit [23] is an improved version of charge qubit by increasing the ratio of EjEjEcEc to the order of $10^{2}$, which reduces the qubit’s sensitivity to charge noise and improves its decoherence time. And it is the most widely used in realizing superconducting quantum computing [24]. According to whether the qubit frequency can be tuned, the qubits can be divided into fixed frequency qubits and tunable frequency qubits. For a fixed frequency qubit, it requires fewer control lines (no DC bias and magnetic flux crosstalk), so it has long coherence time [16,17,18,25]. The disadvantage is that its frequency is not tunable, and the qubit frequencies need to be strictly controlled in the process. On the contrary, the frequencies of frequency-tunable qubits can be tuned by adjusting the magnetic flux flowing through the SQUID structure which consists of two Josephson junctions, allowing more flexibility in the implementation of two qubits. But it requires more control lines which introduce more noise channels, thus the decoherence time is relatively short. Current long-coherence quantum chips are all based on fixed-frequency qubits [25,26,27].

For designing superconducting quantum chips, the set of qubit and readout resonator can be regarded as an open quantum mechanical system, determining the Hamiltonian of a system is the key step in deriving its dynamical system. To quantify the Hamiltonian of a superconducting quantum circuit system, a variety of new quantization methods have been proposed, such as impedance-based black-box quantization, lumped oscillator model and energy participation ratio method [28,29,30], etc. This paper will first introduce the lumped oscillator model method and the energy participation ratio method. Then, based on these two methods, a four-qubit fixed-frequency quantum chip is designed and prepared. Finally, the experimental measurement results will be presented. By comparing the theoretically calculated values and the experimentally measured data, we verify the feasibility of these two methods to design two-dimensional superconducting quantum chips and discuss their advantages and disadvantages.

## 2. Quantization of Superconducting Circuits

### 2.1. Lumped Oscillator Model

The lumped oscillator model (LOM) method is derived based on the quantization of the lumped model. In the LOM method, the distributed microwave circuit is equivalented to a lumped circuit [31], and the computational efficiency is higher than the full-wave method [29]. Its core idea is to divide the physical layout of a quantum processor into disjoint units, each of which can independently extract electrical parameters.

Taking a subsystem coupled to *K* neighbors as an example, the Hamiltonian of the composite system [29,31] is

(1) $${\hat{H}}_{full}={\hat{H}}_{0}+\sum_{n=1}^{K}{\hat{H}}_{n}+\sum_{n=0}^{K-1}\sum_{m=n+1}^{K}{\hat{H}}_{nm},$$

where ${\hat{H}}_{0}$ and ${\hat{H}}_{n}$ are the Hamiltonians of the subsystem and the *n*-th adjacent structure, respectively, and ${\hat{H}}_{nm}$ is the Hamiltonian of the interaction between the *n*-th subsystem and the *m*-th subsystem. The interacting Hamiltonian has the following form [29,31]

(2) $${\hat{H}}_{nm}=\frac{{\hat{Q}}_{n}{\hat{Q}}_{m}}{C_{nm}^{eff}}+\frac{{\hat{\Phi }}_{n}{\hat{\Phi }}_{m}}{L_{nm}^{eff}},$$

where $C_{nm}^{eff}$ and $L_{nm}^{eff}$ represent the effective capacitance and the effective inductance, respectively. ${\hat{\Phi }}_{n}$ and ${\hat{\Phi }}_{m}$ represent the charge operator and the magnetic flux operator, respectively. From Equation (2), it is found that the confirmation of the Hamiltonian requires solving the capacitance and inductance values between the different subsystems. These values can be added to the matrix, which is uniformly expressed by the capacitance matrix. Due to the detailed consideration of the coupling to other subsystems, the method accurately calculates the frequency of the qubit, and its error will be exhibited in the experimental results section.

**Figure 1:**
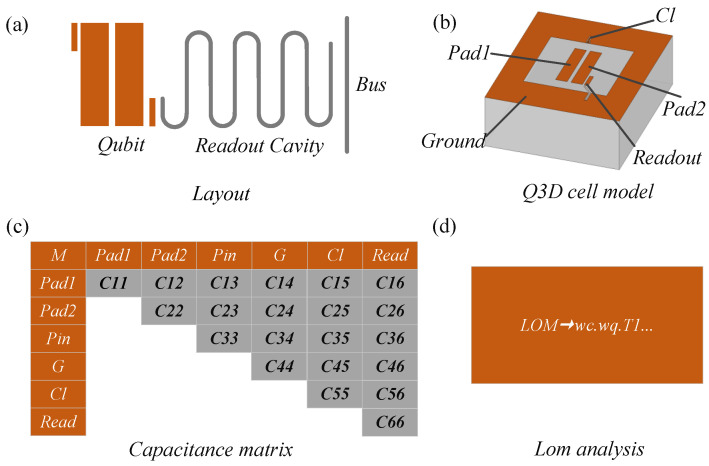
(**a**) Schematic diagram of the physical layout of one qubit coupled to one readout resonator. (**b**) Schematic diagram of the Q3D model of the Transmon qubit. (**c**) Capacitance matrix derived from Q3D simulation. (**d**) LOM analysis.

The entire schematic diagram of the method is shown in Figure 1. The composite system consists of qubits, a coplanar waveguide readout resonator, and a coplanar waveguide transmission bus. The node-set for the qubit unit is $\left\{Pad1,Pad2,Cl,Ground,Readout\right\}$ as shown in Figure 1b. The Maxwell capacitance matrix can be extracted using Ansys Q3D Extractor, as shown in Figure 1c, based on the physical layout of the qubit, shown in Figure 1b. The capacitance matrix is symmetric. The elements in the *i*-th row and the *j*-th column represent the coupling capacitance between the corresponding nodes. The capacitance on the main diagonal is the algebraic sum of the elements in each row. Then, the different capacitance values of the capacitance matrix are brought into the formula for calculation, and the theoretical calculation values of different parameters are obtained.

For the description of a physical system, we need more parameters. In the LOM method, the qubit frequency is determined by the following formula:

(3) $$\omega_{q}=\frac{1}{\sqrt{L_{j}C_{q}}}-E_{c},$$

where $E_{c}$ is the capacitance energy of the superconducting qubit, $L_{j}$ is the inductance of the Josephson junction, and $C_{q}$ is the capacitance of the qubit. The relationship between $E_{c}$ and $C_{q}$ is

(4) $$E_{c}=\frac{e^{2}}{2C_{q}}.$$

The inductance of the Josephson junction can be determined by the magnetic flux quantum and the critical current,

(5) $$L_{j}=\frac{\Phi_{0}}{I_{c}}.$$

In this method, for a quarter-wavelength readout resonator, its equivalent capacitance and inductance are

(6) $$C=\frac{\pi }{4\omega_{r}Z_{c}},L=\frac{1}{{\omega_{c}}^{2}C},$$

respectively, where $\omega_{c}$ is the frequency of the resonator, and $Z_{c}$ represents the characteristic impedance of the resonator. And the relationship between the length and the frequency of the readout cavity is given by

(7) $$\omega_{c}=\frac{c\pi }{2l}\sqrt{\frac{2}{\varepsilon_{r}+1}}.$$

### 2.2. Energy Participation Ratio

The Energy Participation Ratio (EPR) method is a circuit quantization method derived from the first principles. This method transforms the quantization problem of circuits into an issue of determining an energy proportion: determining how much of the energy of mode *m* is stored in element *j* [28], called the energy participation ratio, denoted by $P_{mj}$. This ratio is the key to resolving the Hamiltonian and plays an essential role in the building blocks of the many-body Hamiltonian [28].

First, consider a system of simple Transmon qubits coupled directly to a readout resonator [28]. The Hamiltonian ${\hat{H}}_{full}$ of the system can be divided into linear and nonlinear parts:

(8) $${\hat{H}}_{full}={\hat{H}}_{lin}+{\hat{H}}_{nl},$$

where ${\hat{H}}_{lin}$ is composed of the linear energy part of the Josephson junction and the linear energy correlation term of the resonator, and ${\hat{H}}_{nl}$ is the nonlinear energy correlation term of the Josephson junction. The Hamiltonians of these two parts are:

(9) $${\hat{H}}_{lin}=\hbar \omega_{c}{{\hat{a}}_{c}}^{†}{\hat{a}}_{c}+\hbar \omega_{q}{{\hat{a}}_{q}}^{†}{\hat{a}}_{q},$$

(10) H^nl=−Ejcosφ^j+φ^j2φ^j222,

(11) $${\hat{\varphi }}_{j}=\varphi_{q}\left({\hat{a}}_{q}+{{\hat{a}}_{q}}^{†}\right)+\varphi_{c}\left({\hat{a}}_{c}+{{\hat{a}}_{c}}^{†}\right),$$

where $\omega_{c}$ and $\omega_{q}$ are the angular frequency of the resonator and the eigenmode frequency of the qubit, respectively, related to the linear Hamiltonian, and ${\hat{a}}_{c}$ and ${\hat{a}}_{q}$ are their annihilation operators, respectively. $E_{j}$ is the energy of the Josephson junction. $\varphi_{c}$ and $\varphi_{q}$ are the quantum zero-point fluctuations in the cavity and qubit modes [28], respectively. By running the eigensolver within a certain range through finite element analysis, we can obtain the mixed cavity and qubit modes as well as the eigenfrequencies $\omega_{c}$ and $\omega_{q}$, and finally derive the linear part of the Hamiltonian, namely ${\hat{H}}_{lin}$.

For the nonlinear Hamiltonian ${\hat{H}}_{nl}$, from Equation (10), we need to know the quantum zero-point fluctuations $\varphi_{c}$ and $\varphi_{q}$, which can be calculated from the energy participation ratio. The energy participation $P_{m}$ of the junction in mode *m* is defined as the ratio of the inductive energy stored in the Josephson junction relative to the inductive energy stored in the entire circuit [28]. $P_{m}$ can be calculated from electromagnetic field and is related to quantum zero-point fluctuations as follows [28]:

(12) $${\varphi_{c}}^{2}=p_{c}\frac{\hbar \omega_{c}}{2E_{j}},$$

(13) $${\varphi_{q}}^{2}=p_{q}\frac{\hbar \omega_{q}}{2E_{j}},$$

Therefore, through the energy participation ratio, we can get the description of the total Hamiltonian ${\hat{H}}_{full}$.

In addition, we also need to determine the coupling strength and transition frequency of the system in the actual experiment. The effective Hamiltonian of the system is obtained by approximation:

(14) $$\begin{array}{c} {\hat{H}}_{eff}=\left(\omega_{q}-\Delta_{q}\right){\hat{n}}_{q}+\left(\omega_{c}-\Delta_{c}\right){\hat{n}}_{c}-\chi_{qc}{\hat{n}}_{q}{\hat{n}}_{c}\\ \;\;\;\;\;\;\;\;\;\;\;\;\;\;\;\;\;\;\;\;\;\;\;\;\;\;-\frac{1}{2}\alpha_{q}{\hat{n}}_{q}\left({\hat{n}}_{q}-\hat{1}\right)-\frac{1}{2}\alpha_{c}{\hat{n}}_{c}\left({\hat{n}}_{c}-\hat{1}\right), \end{array}$$

where ${\hat{n}}_{q}={{\hat{a}}_{q}}^{†}{\hat{a}}_{q}$ and ${\hat{n}}_{c}={{\hat{a}}_{c}}^{†}{\hat{a}}_{c}$ represent the cavity and qubit particle number operators, respectively, $\Delta_{q}$ is the “Lamb shift” of the qubit frequency, $\alpha_{q}$ is the anharmonicity of the qubit, and $\chi_{qc}$ is the dispersion shift. The main parameters of the Hamiltonian can be directly calculated from the energy participation ratio,

(15) $$\alpha_{q}=\frac{1}{2}\chi_{qq}={p_{q}}^{2}\frac{\hbar {\omega_{q}}^{2}}{8E_{j}},$$

(16) $$\alpha_{c}=\frac{1}{2}\chi_{cc}=p_{c}^{2}\frac{\hbar \omega_{c}^{2}}{8E_{J}},$$

(17) $$\chi_{qc}=p_{q}p_{c}\frac{\hbar \omega_{q}\omega_{c}}{4E_{J}}.$$

And $0\leq p_{q}\left(p_{c}\right)\leq 1,$$p_{q}+p_{c}=1.$

## 3. Experimental Results

**Figure 2:**
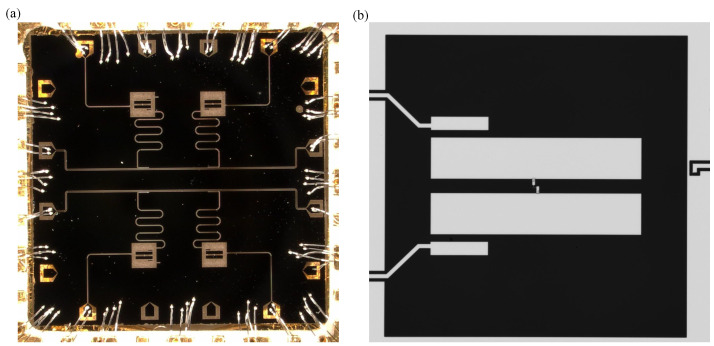
(**a**) Optical microscope photo of the chip. (**b**) Microscope photo of Transmon qubit.

**Figure 3:**
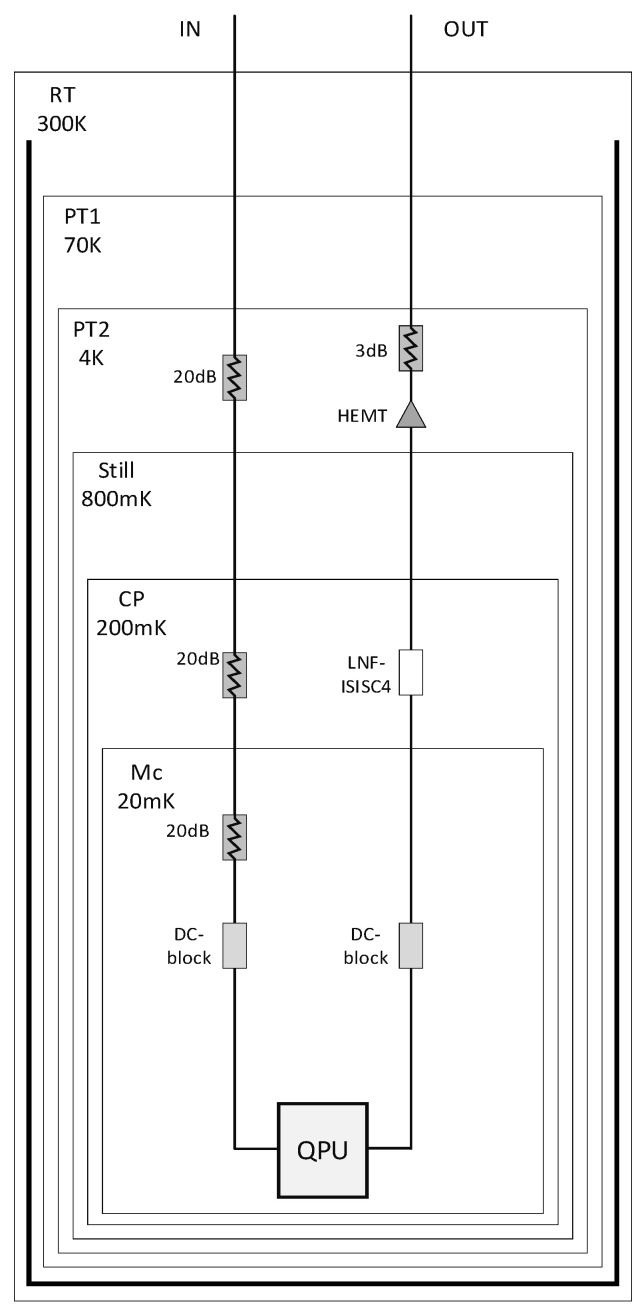
Cryogenic wiring for one of the qubits. Wiring for other qubits is identical.

The chip with an area of $8\times 8\;{\mathrm{mm}}^{2}$ contains four fixed-frequency transmon qubits and two transmission buses. The state of each qubit is readout by a corresponding readout resonator. We evaporated 100 nm aluminum on a 430 µm sapphire substrate by electron beam evaporation and then performed photolithography and etching processes to construct the readout resonator of the chip and the capacitance part of the qubit. We use electron-beam lithography to pattern Al/AlOx/Al Josephson junctions. Figure 2a is a photo of the entire chip under the microscope. Figure 2b shows the optical picture of a transmon qubit. The cryogenic wiring for the chip is shown in Figure 3. The device was tested in a dilution refrigerator at temperature below 20 mK.

By comparing the theoretically calculated results with the experimentally measured data, we find that the calculation errors of the LOM method for the qubit frequency, the resonator frequency, and the room temperature resistance are all within 10%. The qubit frequency is calculated by Equation (3), where the qubit capacitance can be calculated from the capacitance matrix. In this example, the capacitance matrix of the qubit is shown in Table 1. The diagonal elements of the capacitance matrix are the sum of the capacitances of a node and other nodes. The capacitance of a qubit is formed by adding the capacitance of the junction to the capacitance of the other parts,

(18) $$C_{q}=tCSq+C_{J},$$

where $C_{J}$ is the junction capacitance and tCSq is the total capacitance between other coupling structures,

(19) $$tCSq=Cs+\frac{C1S\times C2S}{C1S+C2S}.$$

where $C_{S}$ is the capacitance between Pad1 and Ground, C1S is the sum of the capacitance between Ground and Cl, the capacitance between Ground and Pad2, and the capacitance between Ground and Readout pin. C2S is the capacitance between Pad1 and Pad2, and the capacitance between Pad1 and Readout pin. Since the calculation of the qubit frequency takes into account the capacitance of each coupling structure around the bit, the calculation for the qubit frequency is accurate, with an error of only 0.2%, as shown in Table 2.

The theoretically calculated values and exprimentally measured data are summarized in the tables below.

**Figure 4:**
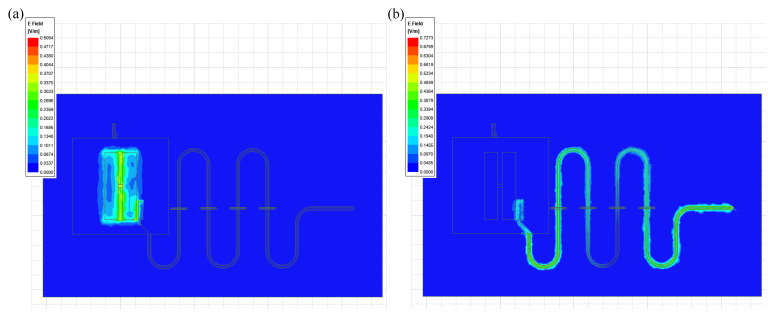
(**a**) Electric field distribution of qubit mode. (**b**) Electric field distribution of cavity mode.

The energy participation ratios are obtained by simulating the electric field, then the important parameters of the Hamiltonian are further calculated using the participation ratios. As shown in the system discussed in Section 2.2, the model is a Transmon qubit coupled to a resonant cavity. In the calculation process, we used the eigenmode solver of HFSS. The Josephson junction is equivalent to a rectangular inductor, and its inductance value is set to 13 nH. Figure 4 shows the electric field distributions in the qubit mode and the cavity mode, respectively. We select qubit frequency and anharmonicity for error analysis. In the EPR method, the qubit frequency error is about 16.8%, and the anharmonicity error is 13.5% as shown in Table 3.

In terms of data diversity, the LOM method provides more parameter data, such as the equivalent inductance, critical current, and room temperature resistance of the Josephson junction.

## 4. Discussion

The essence of the lumped oscillation model is to convert the traditional microwave distributed circuit into a lumped circuit, extract the capacitance matrix by using Q3D, and then calculate the essential parameters related to the determination of the system Hamiltonian. Its advantage is that it is more computationally efficient. The reason for the high agreement between the calculated value of the qubit frequency and the experimental result is that the coupling between the subsystem and other systems is fully considered, and the capacitance matrix is accurate. The coupling capacitance with the subsystem is simulated to obtain a more precise value of the qubit capacitance. According to the calculation formula of the qubit frequency, the theoretical calculation value with an error of 0.2% from the experiment is obtained. In addition, the method also calculates the decoherence time under different dissipative channels (readout resonator, inter-qubit coupling cavity, control line), and finally gives the upper limit of T1 time.

The EPR method transforms the problem of determining the Hamiltonian of a superconducting quantum system into a parameter calculation problem related to the energy participation ratio. In this process, the finite element eigenmode solver simulates the qubit frequency and cavity frequency. Its advantage is that the method is more suitable for multi-junction circuits. But in this experiment, there was a 12% deviation in the qubit frequency. The Josephson junction is equivalent to a rectangular inductor in the HFSS eigenmode solver. However, we found that when the magnitude of the equivalent inductance of the rectangle is significantly changed, the eigensolver of HFSS does not show a relatively obvious frequency change, which is an unreasonable phenomenon. Therefore, there is a specific error in this approximation during this simulation.

## 5. Conclusions

In this paper, we fabricated a four-qubit fixed-frequency quantum chip designed based on the LOM and EPR method. By comparing the theoretically calculated values of some parameters of the chip using these two methods with the corresponding experimental data, we find that the LOM method is a more efficient method in this experiment. The reasons for the relatively sizeable relative errors of the EPR method are discussed. From the previous data, it can be concluded that when designing simpler quantum circuits, such as small-scale fixed-frequency quantum chips, the LOM method can reasonably approximate the important parameters of qubits. Besides, this method has more approximate parameters. For multi-junction circuits, the EPR method is more accurate. However, the approximate method of Josephson junction in the finite element eigenmode analysis still needs further research. Moreover, the EPR method can be used to study the dissipation of different devices, so we guess that the EPR method is expected to be extended to the study of the decoherence time. Finally, the mathematical description of the energy participation ratio (or energy dissipation rate) and the qubit decoherence time can be obtained.

## Figures and Tables

**Table 1:** This is a capacitance matrix, the unit is fF.

Matrix	Readout	Cl	Ground	Pad1	Pad2
Reaeout	49.53	−0.008	−33.57	−13.51	−1.63
Cl	−0.008	−16.15	−15.64	−0.169	−0.257
Ground	−33.57	−15.64	200.25	−43.21	−47.96
Pad1	−13.51	−0.169	−43.21	95.07	−35.17
Pad2	−1.63	−0.257	−47.96	−35.17	88.24

**Table 2:** Comparison between the theoretically calculated (c) and experimentally measured (m) values of the LOM method of qubit parameters. The percentages in the third row are the error values, i.e., e=|(c−m)/c|.

LOM	$\omega_{q}\left(\mathrm{GHz}\right)$	$\omega_{c}\left(\mathrm{GHz}\right)$	$\alpha \left(\mathrm{MHz}\right)$	$R_{n}\left(k\Omega \right)$	$T_{1}\left(\mu s\right)$
*c*	4.731	6.5	−286	11.3	135
*m*	4.732	5.9	−350	11.0	31
*e*	0.2%	0.9%	22%	2.5%	-

**Table 3:** Comparison between the theoretically calculated (c) and experimentally measured (m) of the EPR method for qubit parameters. The percentages in the third row are the error values, i.e., e=|(c−m)/c|.

EPR	$\omega_{q}\left(\mathrm{GHz}\right)$	$\alpha \left(\mathrm{MHz}\right)$
*c*	5.690	−405
*m*	4.732	−350
*e*	16.8%	13.5%

## Data Availability

The data that support the findings of this study are available from the corresponding author upon reasonable request.
